# Association of *ABCB1* and *MTHFR* genetic polymorphisms with rheumatoid arthritis susceptibility in a Chinese population

**DOI:** 10.3389/fmed.2025.1660473

**Published:** 2025-10-10

**Authors:** Yanfei Chen, Jirong Wu, Qiaomei Dong, Juan Li

**Affiliations:** ^1^Department of Rheumatology and Immunology, The First Hospital of Lanzhou University, Lanzhou, China; ^2^Dong Fureng Economic & Social Development School, Wuhan University, Wuhan, China; ^3^Central Laboratory, The First Hospital of Lanzhou University, Lanzhou, China

**Keywords:** ATP binding cassette-related transporter B1, methylene tetrahydrofolate reductase, arthritis, rheumatoid, polymorphism, single nucleotide, Chinese

## Abstract

**Objective:**

The present study aimed to investigate the association of the *ATP-binding cassette subfamily B member 1 (ABCB1) 3435C/T* and *methylenetetrahydrofolate reductase (MTHFR) 677C/T* and *1298A/C* polymorphisms with susceptibility to RA and RA subtype.

**Methods:**

We enrolled 85 RA patients and 45 healthy individuals in this study. Genotyping for *ABCB1 3435C/T*, *MTHFR 677C/T* and *1298A/C* was performed using fluorescence *in situ* hybridization technology (FISH).

**Results:**

No significant differences in the genotype and allele frequency distributions of *ABCB1 3435C/T*, *MTHFR 1298A/C* or *MTHFR677C/T* were detected between patients with RA and healthy individuals (*p > 0.05*). Logistic regression analysis, after adjusting for sex and age, showed that the *MTHFR677C/T TT* genotype was associated with increased susceptibility to RA compared with the *CC* genotype (*TT versus CC*, *p = 0.034*, OR = 2.316; 95%CI = 1.067–5.029); however, after Bonferroni correction, the association between the MTHFR677C/T TT genotype and RA susceptibility was no longer significant. That *MTHFR 677C/T* genotype frequency (*CT versus CC*, *p = 0.040*, OR = 6.504; 95% CI = 1.087–38.935) and dominant model (*CT+TT versus CC*, *p = 0.025*, OR = 6.556; 95% CI = 1.272–33.799) was associated with ACPA status among RA patients; and that *MTHFR 677C/T* genotype frequency (*TT versus CC*, *p = 0.044*, OR = 2.171; 95% CI = 1.020–4.623) and codominant model (*CC versus CT versus TT*, *p = 0.026*, OR = 2.059; 95% CI = 1.089–3.891) were associated with RF status among RA patients; Similarly, the associations observed in ACPA-positive and RF-positive subgroups also lost significance after Bonferroni correction.

**Conclusion:**

The *MTHFR 677C/T* polymorphism may be associated with susceptibility to RA and RA subtypes; *ABCB1 3435C/T* and *MTHFR 1298A/C* were not associated with the susceptibility to RA or RA subtypes.

## Background

Rheumatoid arthritis (RA) is one of the most common autoimmune diseases and is characterized by chronic inflammation of joint synovial membranes and by pannus formation. Its main manifestation in the symmetrical small joints is often accompanied by extra-articular manifestations, such as chronic anemia, vasculitis, and interstitial pneumonia (1). The disease affects approximately 0.5–1.0% of the adult population worldwide and is more frequent in women (3:1) (2). The etiology and pathogenesis of RA are not yet clearly defined, but it is generally believed that genetic factors play an important role in the pathogenesis of RA. To date, more than 100 genetic susceptibility loci for RA have been identified through large-scale population studies. These genetic susceptibility loci can be classified as *human leukocyte antigen (HLA)* class and *non-HLA* loci (3). The *HLA* loci explains only one third of the entire genetic effect (4). Therefore, a large number of *non-HLA* genetic susceptibility loci must remain to be discovered.

Methotrexate (MTX), as a first-line traditional synthetic anti-rheumatism drug (DMARD), has the advantages of clear efficacy and low price, so it plays a core role in the treatment of RA in China (5). As a competitive inhibitor of dihydrofolate reductase (DHFR), MTX affects the synthesis of one carbon unit by inhibiting the activity of DHFR. In addition, due to its effect on the folic acid pool in cells, MTX can also affect the activity of methylene tetrahydrofolate reductase and eventually interfere with the normal metabolism of folic acid to achieve the therapeutic effect. MTX outflow from cells is accomplished by ABC family transporters, among which *ABCB1* plays an important role (6, 7). Therefore, genetic variation in the *MTHFR* and *ABCB1* genes is closely related to the efficacy and toxicity of MTX in RA patients (8, 9). Recent studies have shown that some genes with roles in drug metabolism are also related to disease phenotypes (10). Studies have found that the P-glycoprotein (P-gp) encoded by the ABCB1 gene is involved in the transport of cytokines (e.g., IL-2, IL-4, IFN-*γ*, TNF-*α*), and its decreased function may lead to the accumulation of proinflammatory cytokines, thereby exacerbating RA inflammation (11). Additionally, studies have shown that *MTHFR*-a key enzyme in folate metabolism-exhibits decreased activity that results in elevated homocysteine levels, and hyperhomocysteinemia is associated with the pathogenesis of RA (12, 13).

The human *ABCB1* gene is located on the long arm of chromosome 7 at 7Q21.1 and encodes the 170 kDa plasma membrane protein P-gp, which is a member of the ABC transporter superfamily. Only 15 single nucleotide polymorphisms (SNPs) were identified in the first analysis of the exon sequences of the *ABCB1* gene, but this number later increased to more than 50 SNPs and insertion or deletion polymorphisms (14, 15). Studies in populations of different races showed that the most common SNPs in *ABCB1* are *3435C/T*, *2677G/T/A/C*, and *1236C/T* (16). Previous studies have shown that the *ABCB1 3435C/T* gene polymorphism is related to the P-gp expression level and substrate uptake and that the P-gp expression level of CC genotype carriers is significantly lower than that of TT genotype carriers (17). Muralidharan et al. (11) studied 336 RA patients in South India and found that RA patients carrying the *ABCB1 3,435 T* allele had a higher EULAR disease activity index. The *CT* genotype is closely related to the degree of joint destruction in RA and is a protective factor against infection in RA patients. However, Boughrara et al. (18) found that the *ABCB1 3435C/T* gene polymorphism was not associated with RA susceptibility in 110 African RA patients. This difference in conclusions may be due to differences in research methods, sample size, and the region of origin and ethnic backgrounds of the study populations, which can generate uncertainties and inconsistencies in the results of different studies.

The human *MTHFR* gene is located on chromosome 1p36.3 and has a length of 1980 bp. The *MTHFR* gene sequence includes multiple SNPs that can affect the synthesis and metabolism of folic acid *in vivo* by changing its enzymatic activity. The study of Frosst et al. (19) showed that the activity of the heterozygous gene product of *MTHFR 677CT* was approximately 65% of that of the wild-type homozygous product, while the activity of the mutant homozygous gene product decreased significantly to only 30%. The activity of the gene product of *MTHFR 1298CC* mutant homozygotes was approximately 60% of that of wild-type homozygotes (20). In recent years, the *MTHFR* gene polymorphism has been found to be closely associated with the incidence of systemic lupus erythematosus (SLE) (21). In recent years, some studies have analyzed the association between *MTHFR* gene polymorphisms (*C677T* and *A1298C*) and genetic susceptibility to RA in different ethnic populations, but the results have been contradictory (22, 23). In this study, fluorescence *in situ* hybridization (FISH) was used to detect the polymorphisms *ABCB13435C/T*, *MTHFR 677C/T*, and *MTHFR 1298A/C* in RA patients and healthy controls in order to analyze the correlation between *ABCB1 3435C/T*, *MTHFR 677C/T*, and *MTHFR 1298A/C* and RA susceptibility and RA subtype and to explore the possible roles of these polymorphisms in the pathogenesis of RA.

## Materials and methods

### Patients and healthy controls

A total of 85 RA patients (68 women and 17 men; average age 54.69 ± 13.56 years) were selected from the outpatient and inpatient departments of Lanzhou University First Hospital from August 2016 to December 2018. All cases met the revised RA classification criteria in ACR1987 years (24) or the classification criteria developed by the ACR and EULAR in 2010 (25). Patients with systemic lupus erythematosus, inflammatory myopathy, systemic sclerosis and other connective tissue diseases were excluded. All cases with available demographic data and laboratory investigation included the presence of rheumatoid factor (RF), and anti-Citrulinated Protein Antibody (anti-CCP). All samples were immediately used for subsequent analysis after collection.

The healthy control group consisted of healthy individuals who underwent physical examinations at the Physical Examination Center of the First Hospital of Lanzhou University during the same period and included a total of 48 cases, including 28 women and 20 men, with an average age of 43.31 ± 14.73 years. Each participant’s family genetic history and medical history were collected at the outpatient Department of Rheumatology and Immunology and a health examination was conducted; no abnormalities in rheumatoid factors, C-reactive protein, erythrocyte settlement rate, or X-ray bone scan were found in any case. All samples were immediately used for subsequent analysis after collection. This study was approved by the Medical Ethics Committee of Lanzhou University First Hospital, and all study subjects signed an informed consent form. The general characteristics of RA patients and the healthy control group are shown in Table 1.

**Table 1:** General data of RA patients and healthy controls.

Variable	RA patients
Gender	
Female	68 (80.0%)
Male	17 (20.0%)
Age	54.69 ± 13.56
Occupation	
Intellectual	12 (14.1%)
Workman	17 (20.0%)
Peasantry	33 (38.8%)
Inoccupation	23 (27.1%)
Disease duration	
≦6 months	8 (9.4%)
6–24 months	10 (11.8%)
≧24 months	67 (78.8%)
ACPA	
ACPA-positive	20 (44.4%)
ACPA-negative	25 (55.6%)
RF	
RF-positive	66 (86.8%)
RF-negative	10 (13.2%)

### Genotyping

For all subjects, 2 mL of venous blood was collected using EDTA anticoagulant tubes and gently inverted to mix thoroughly. Ammonium chloride solution was diluted with sterile water for injection at a ratio of 1:9 to prepare the working solution. In a 1.5 mL plastic centrifuge tube, 1,000 μL of the working solution was added, followed by 150 μL of the mixed blood sample to be tested. The tube was inverted to mix, and then allowed to stand at room temperature for 5 min. The sample was placed in a centrifuge and centrifuged at 3000 rpm for 5 min (centrifugal radius: 3 cm). The supernatant was discarded, and the enriched white blood cell pellet was obtained at the bottom of the centrifuge tube. To the centrifuge tube containing the enriched white blood cells, 100 μL of PHARM-GENE SNP Analysis Preservation Solution (trade name: Yaojinbao Reagent) was added. The tube was vortexed using a vortex mixer to mix uniformly, then incubated at room temperature for 20–30 min, with gentle inversion to mix twice during this period. A volume of 1.0–1.5 μL of the white blood cell preservation solution was aspirated and added separately to the PHARM-GENE 200SNP Analysis Sample Processing Reagents (for *ABCB1 3435 T/C, MTHFR 677C/T,* and *MTHFR 1298A/C* loci; trade name: Yaojinfen Reagent). The mixture was vortexed to mix, subjected to brief centrifugation, and then loaded onto the instrument for detection.

### Statistical analysis

Statistical processing was performed using SPSS 19.0 statistical software (SPSS Inc., Chicago, IL, United States) for data analysis. In this study, the age of RA patients and healthy controls was continuous data that conformed to a normal distribution and homogeneity of variance. Therefore, it was expressed as the mean±standard deviation (*x̅* ± s), and the inter-group comparison was performed using an independent samples *t*-test. The analysis of the distributions of genotype frequencies and allele frequencies of the three SNP loci in the *ABCB1* and *MTHFR* genes between the RA group and the healthy control group was first conducted using the chi square (*χ*^2^) test. The strength of the statistical association was expressed as the odds ratio (OR) and 95% confidence interval (CI). Moreover, logistic regression analysis was utilized to calculate the OR values and 95% CIs after adjusting for age and gender. The Hardy–Weinberg equilibrium (HWE) law was used to analyze whether the distribution of genotypes at each SNP locus in the healthy control population conforms to the genetic equilibrium law. The SHEsis online software was used to perform linkage disequilibrium and haplotype analysis. Differences were considered statistically significant at *p* < 0.05. Meanwhile, to minimize the possibility of false positive results that may occur during multivariate association analysis, we also performed strict Bonferroni correction on the *p*-values <0.05.

## Results

### General data of RA patients and healthy controls

A total of 85 RA patients were enrolled, including 68 women (80.0%) and 17 men (20.0%), ranging in age from 18 to 87 years (average 54.69 ± 13.56 years). The healthy control group included 48 individuals, 28 females (58.3%) and 20 males (41.7%), with a mean age of 43.31 ± 14.73 years. The sex composition and mean age were significantly different between the RA patients and healthy controls groups (*p < 0.05*). Rheumatoid factor (RF) data from 76 RA patients and anti-citrulline protein antibody (ACPA) data from 45 patients are shown in Table 1.

### Hardy–Weinberg equilibrium test for genotype distribution of SNP loci

To test whether the genotype frequency distributions of the SNP loci in the *ABCB1* and *MTHFR* genes conformed to HWE in the healthy control group, the analysis results showed that the genotype distributions of the *ABCB1 3435C/T, MTHFR 677C/T, and MTHFR 1298A/C* loci in the control group all conformed to HWE equilibrium (with all *p*-values *>0.05*), as shown in Table 2.

**Table 2:** HWE test results for SNPs loci of the studied genes in the healthy control group.

Gene SNPs	Genotype	Observed frequency	Expected frequency	*χ* ^2^	*p* value
	CC	18	18		
*ABCB13435C/T*	CT	23	23	0.000	1.000
	TT	7	7		
	CC	12	12		
*MTHFR677C/T*	CT	23	24	0.061	0.970
	TT	13	12		
	AA	36	36		
*MTHFR1298A/C*	AC	11	11	0.000	1.000
	CC	1	1		

### Genotype and allele frequencies of SNPs in the *ABCB1* and *MTHFR* genes and their correlations with susceptibility to RA

The *ABCB1 3435C/T* and *MTHFR 1298A/C* genotypes and allele distribution frequencies did not differ between RA and healthy controls (*p > 0.05*). The RA group also showed no significant difference from the control group in terms of the genotype and allele frequencies of *MTHFR 677C/T* (*p > 0.05*). Since the RA group and the healthy control group exhibited statistically significant differences in gender composition and age distribution, with reference to the analytical approaches used by Zhao et al. (26), Liu et al. (27), and Chen et al. (28), Logistic regression analysis was employed to adjust for gender and age. After adjustment, it was found that the frequency of the TT genotype exhibited a statistically significant difference when compared with that of the CC genotype (*p* = 0.034, OR = 2.316, 95% CI = 1.067–5.029). However, after correction using the Bonferroni method, the association between the TT genotype of *MTHFR* 677C/T and RA disappeared. Additionally, no statistically significant associations were observed under the three genetic models (dominant, recessive, and codominant models), as shown in Tables 3, 4.

**Table 3:** Allele and genotype frequencies of SNPs in the *ABCB1* and *MTHFR* genes in RA patients and healthy controls.

SNPs	Genotype/Allele	RA patients (*N* = 85)	Healthy controls (*N* = 48)	Unadjustment			Adjustment with sex and age	
				*χ*^2^ value	*P* value	OR (95%CI)	*P* value	OR (95%CI)
*ABCB13435C/T*	CC	31 (36.47%)	18 (37.5%)			1.00		
	CT	35 (41.18%)	23 (47.9%)	1.268	0.260	0.561 (0.203–1.545)	0.169	2.165 (0.721–6.502)
	TT	19 (22.4%)	7 (14.6%)	0.736	0.391	0.635 (0.224–1.801)	0.666	1.145 (0.618–2.123)
	T	73 (42.9%)	37 (38.5%)	0.490	0.484	1.200 (0.720–2.001)		
	C	97 (57.1%)	59 (61.5%)					
*MTHFR677C/T*	CC	28 (32.9%)	12 (25.0%)			1.00		
	CT	43 (50.6%)	23 (47.9%)	0.265	0.607	0.801 (0.344–1.865)	0.422	1.472 (0.573–3.783)
	TT	14 (16.5%)	13 (27.1%)	2.270	0.132	0.462 (0.168–1.272)	0.034	2.316 (1.067–5.029)
	C	99 (58.2%)	47 (49.0%)	2.132	0.144	1.454 (0.879–2.404)		
	T	71 (41.8%)	49 (51.0%)					
*MTHFR1298A/C*	AA	56 (65.9%)	36 (75.0%)			1.00		
	AC	27 (31.8%)	11 (22.9%)	1.208	0.272	1.578 (0.697–3.570)	0.088	0.428 (0.161–1.135)
	CC	2 (2.4%)	1 (2.1%)	0.041	0.839	1.286 (0.112–14.702)	0.852	0.889 (0.257–3.074)
	A	139 (81.8%)	83 (86.5%)	0.979	0.322	1.424 (0.705–2.874)		
	C	31 (18.2%)	13 (13.5%)					

**Table 4:** Three genetic models (dominant/recessive/codominant) of SNPs in the *ABCB1* and *MTHFR* genes in RA patients and healthy controls.

SNPs	Genetic models	Genotype	RA patients (*N* = 85)	Healthy controls (*N* = 48)	Unadjustment			Adjustment with sex and age	
					*χ*^2^ value	*P* value	OR (95%CI)	*P* value	OR (95%CI)
*ABCB13435C/T*	Dominant	TT + CT	54 (63.5%)	30 (62.5%)	0.014	0.906	0.957 (0.460–1.990)	0.575	0.784 (0.334–1.840)
		CC	31 (36.47%)	18 (37.5%)					
	Recessive	TT	19 (22.4%)	7 (14.6%)	1.177	0.278	0.593 (0.229–1.534)	0.290	1.749 (0.622–4.921)
		CT + CC	66 (77.6%)	41 (85.4%)					
	Codominant	TT vs. TC vs. CC	19/35/31	7/23/18	1.276	0.528		0.820	1.065 (0.619–1.832)
*MTHFR677C/T*	Dominant	CT + TT	57 (67.1%)	36 (75.0%)	0.920	0.338	0.679 (0.307–1.502)	0.210	1.785 (0.721–4.421)
		CC	28 (32.9%)	12 (25.0%)					
	Recessive	TT	14 (16.5%)	13 (27.1%)	2.135	0.144	0.531 (0.225–1.250)	0.117	2.220 (0.819–6.021)
		CC + CT	71 (83.5%)	35 (72.9%)					
	Codominant	CC vs. CT vs. TT	28/43/14	12/23/13	2.389	0.303		0.088	1.671 (0.926–3.017)
*MTHFR1298A/C*	Dominant	AC + CC	29 (34.1%)	12 (25.0%)	1.196	0.274	1.554 (0.703–3.431)	0.098	0.457 (0.180–1.156)
		AA	56 (65.9%)	36 (75.0%)					
	Recessive	CC	2 (2.4%)	1 (2.1%)	0.010	0.920	0.833 (0.078–9.999)	0.968	0.951 (0.081–11.159)
		AC + AA	83 (97.6%)	47 (97.9%)					
	Codominant	AA vs. AC vs. CC	56/27/2	36/11/1	1.219	0.544		0.146	0.538 (0.234–1.240)

### Genotype and allele frequencies of SNPs in the *ABCB1* and *MTHFR* genes and their correlations with RA subtypes (ACPA, RF)

Since the RA group and the healthy control group exhibited statistically significant differences in gender composition and age distribution, with reference to the analytical approaches used by Zhao et al. (26), Liu et al. (27), and Chen et al. (28), Logistic regression analysis was employed to adjust for gender and age. After adjustment, with the CC genotype as the reference, the ACPA-positive RA group showed statistically significant differences in the CT genotype and dominant model (CT + TT) of *MTHFR* 677C/T when compared with the healthy control group [(*p* = 0.040, OR = 6.504, 95% CI = 1.087–38.935) and (*p* = 0.025, OR = 6.556, 95% CI = 1.272–33.799)]. However, after correction using the Bonferroni method, the associations between the CT genotype and dominant model (CT + TT) of *MTHFR* 677C/T and ACPA-positive RA disappeared. Additionally, the RF-positive RA group exhibited statistically significant differences in the TT genotype and codominant model (CC vs. CT vs. TT) of *MTHFR* 677C/T when compared with the healthy control group [(*p* = 0.044, OR = 2.171, 95% CI = 1.020–4.623) and (*p* = 0.026, OR = 2.059, 95% CI = 1.089–3.891)]. Nevertheless, after correction via the Bonferroni method, the associations between the TT genotype and codominant model (CC vs. CT vs. TT) of *MTHFR* 677C/T and RF-positive RA vanished. For the *ABCB1 3435C/T* and *MTHFR 1298A/C* loci, no statistically significant difference was found (*p > 0.05*) for any of the different genotype frequencies, allele frequencies, or genetic models in the comparison of the RA and control groups, as shown in Tables 5–8.

**Table 5:** Genotype of SNPs in the *ABCB1* and *MTHFR* genes and their correlations with RA (ACPA) subtypes.

SNPs	Genotype	ACPA+RA vs. ACPA-RA		ACPA+RA vs. Healthy controls		ACPA-RA vs. Healthy controls	
		*P* value	95%CI	*P* value	OR (95%CI)	*P* value	OR (95%CI)
*ABCB13435C/T*	CC						
	CT	0.560	1.765 (0.261–11.938)	0.664	0.664 (0.105–4.208)	0.599	1.506 (0.328–6.926)
	TT	0.694	1.238 (0.427–3.590)	0.160	0.243 (0.034–1.752)	0.445	0.673 (0.243–1.861)
*MTHFR677C/T*	CC						
	CT	0.408	0.547 (0.131–2.284)	0.040	6.504 (1.087–38.935)	0.340	1.961 (0.492–7.809)
	TT	0.808	0.895 (0.365–2.193)	0.056	4.396 (0.965–20.029)	0.366	1.577 (0.587–4.232)
*MTHFR1298A/C*	AA						
	AC	0.545	0.647 (0.158–2.646)	0.612	0.677 (0.150–3.062)	0.229	0.449 (0.122–1.652)

**Table 6:** Three genetic models (dominant/recessive/codominant) of SNPs in the *ABCB1* and *MTHFR* genes and their correlations with RA (ACPA) subtypes.

SNPs	Genetic models	Genotype	ACPA+RA vs. ACPA-RA		ACPA+RA vs. Healthy controls		ACPA-RA vs. Healthy controls	
			*P* value	95%CI	*P* value	OR (95%CI)	*P* value	OR (95%CI)
*ABCB13435C/T*	Dominant	CT + TT	0.907	0.926 (0.256–3.345)	0.252	0.430 (0.102–1.824)	0.293	0.513 (0.148–1.777)
		CC						
	Recessive	TT	0.532	1.792 (0.288–11.165)	0.517	0.544 (0.086–3.429)	0.885	1.147 (0.263–4.994)
		CT + CC						
	Codominant	TT vs. CT vs. CC	0.806	1.120 (0.454–2.761)	0.253	0.551 (0.199–1.530)	0.539	0.770 (0.334–1.775)
*MTHFR677C/T*	Dominant	CT + TT	0.418	0.584 (0.159–2.146)	0.025	6.556 (1.272–33.799)	0.297	2.005 (0.542–7.411)
		CC						
	Recessive	TT	0.888	0.888 (0.170–4.632)	0.469	1.780 (0.374–8.463)	0.590	1.446 (0.378–5.526)
		CC + CT						
	Codominant	CC vs. CT vs. TT	0.536	0.757 (0.314–1.827)	0.064	2.565 (0.947–6.948)	0.332	1.502 (0.661–3.414)
*MTHFR1298A/C*	Dominant	AC + CC	0.477	0.603 (0.149–2.435)	0.749	0.786 (0.181–3.423)	0.222	0.463 (0.135–1.593)
		AA						
	Codominant	AA vs. AC vs. CC	0.415	0.578 (0.154–2.161)	0.943	0.954 (0.260–3.493)	0.270	0.556 (0.196–1.576)

**Table 7:** Genotype of SNPs in the *ABCB1* and *MTHFR* genes and their correlations with RA (RF) subtypes.

SNPs	Genotype	RF + RA vs. RF-RA		RF + RA vs. Healthy controls		RF-RA vs. Healthy controls	
		*P* value	95%CI	*P* value	OR (95%CI)	*P* value	OR (95%CI)
*ABCB13435C/T*	CC						
	CT	0.850	0.783 (0.062–9.821)	0.262	1.931 (0.611–6.102)	0.558	2.464 (0.120–50.462)
	TT	0.467	0.638 (0.190–2.141)	0.608	1.199 (0.600–2.396)	0.426	0.586 (0.157–2.187)
*MTHFR677C/T*	CC						
	CT	0.560	1.601 (0.328–7.805)	0.125	2.272 (0.797–6.478)	0.242	2.734 (0.508–14.711)
	TT	0.690	1.291 (0.368–4.524)	0.044	2.171 (1.020–4.623)	0.528	1.622 (0.361–7.284)
*MTHFR1298A/C*	AA						
	AC	0.616	0.687 (0.158–2.987)	0.086	0.414 (0.151–1.133)	0.095	0.229 (0.041–1.293)

**Table 8:** Three genetic models (dominant/recessive/codominant) of SNPs in the *ABCB1* and *MTHFR* genes and their correlations with RA (RF) subtypes.

SNPs	Genetic models	Genotype	RF + RA vs. RF-RA		RF + RA vs. Healthy controls		RF-RA vs. Healthy controls	
			*P* value	95%CI	*P* value	OR (95%CI)	*P* value	OR (95%CI)
*ABCB13435C/T*	Dominant	CT + TT	0.886	1.112 (0.262–4.715)	0.778	0.879 (0.357–2.159)	0.103	0.170 (0.020–1.433)
		CC						
	Recessive	TT	0.500	0.462 (0.049–4.368)	0.341	1.721 (0.562–5.271)	0.736	0.674 (0.068–6.668)
		CC + CT						
	Codominant	TT vs. TC vs. CC	0.797	0.879 (0.331–2.338)	0.736	1.107 (0.613–1.999)	0.207	0.384 (0.087–1.702)
*MTHFR677C/T*	Dominant	CT + TT	0.586	1.505 (0.346–6.555)	0.052	2.790 (0.991–7.857)	0.173	3.144 (0.605–16.341)
		CC						
	Recessive	TT	0.989	0.983 (0.095–10.212)	0.091	2.435 (0.868–6.832)	0.416	2.604 (0.260–26.084)
		CC + CT						
	Codominant	CC vs. CT vs. TT	0.691	1.259 (0.404–3.922)	0.026	2.059 (1.089–3.891)	0.170	2.337 (0.695–7.860)
*MTHFR1298A/C*	Dominant	AC + CC	0.644	0.706 (0.161–3.086)	0.090	0.436 (0.167–1.138)	0.117	0.255 (0.046–1.408)
		AA						
	Codominant	AA vs. AC vs. CC	0.711	0.769 (0.191–3.091)	0.131	0.518 (0.220–1.216)	0.188	0.378 (0.089–1.607)

### Linkage disequilibrium and haplotype analysis of two SNP loci in the *MTHFR* gene

There was strong linkage disequilibrium between the two SNP loci (*677C/T* and *1298A/C*) of the *MTHFR* gene in the RA group and the healthy control group (D′ = 1.000, r^2^ = 0.163). A total of 4 haplotypes (CA, CC, TA, TC) were constructed for the two SNP loci of the *MTHFR* gene, but there was no statistically significant difference in these 4 haplotypes between the RA group and the healthy control group (χ^2^_total_ = 2.324, *p* = 0.312, df = 2), see Table 9.

**Table 9:** Haplotype analysis of *MTHFR* in RA patients and healthy controls.

Haplotype	RA patients	Healthy controls	*χ*^2^ value	OR (95% CI)	*P* value
CA	68.00	34.00	0.545	1.216 (0.724 ~ 2.042)	0.460
CC	31.00	13.00	0.979	1.424 (0.705 ~ 2.874)	0.322
TA	71.00	49.00	2.133	0.688 (0.416 ~ 1.138)	0.144
TC	0.00	0.00	–	–	–

## Discussion

This study aims to investigate the association between the polymorphic loci of *ABCB1* gene *3435C/T*, *MTHFR* gene *677C/T* and *1298A/C*, and the susceptibility to RA as well as its serological subtypes (ACPA-positive, RF-positive) in the population of Gansu region, China. FISH technology was employed for genotyping of 85 RA patients and 48 healthy controls, and multivariate Logistic regression analysis was used to adjust for confounding factors such as gender and age.

The main findings of this study are as follows: The *ABCB1 3435C/T* and *MTHFR 1298A/C* loci showed no significant association with overall RA or its subtypes. After adjusting for gender and age, the *TT* genotype of the *MTHFR 677C/T* locus was significantly associated with the overall susceptibility to RA (*p* = 0.034, OR = 2.316, 95% CI: 1.067–5.029). Further subgroup analysis revealed that in ACPA-positive RA patients, the *CT* genotype and dominant model (*CT+TT*) of this locus were significantly associated with disease risk [(*p* = 0.040, OR = 6.504, 95% CI: 1.087–38.935) and (*p* = 0.025, OR = 6.556, 95% CI: 1.272–33.799)]; in RF-positive patients, the *TT* genotype and codominant model also showed significant associations [(*p* = 0.044, OR = 2.171, 95% CI: 1.020–4.623) and (*p* = 0.026, OR = 2.059, 95% CI: 1.089–3.891)].

There are few correlation studies of genetic susceptibility to SNP and RA, and only a few studies have reached very inconsistent conclusions, which may be related to the population, region, and sample size included in the study. Boughrara et al. (10) reported that the *C/T* gene polymorphism of *ABCB1 3,435* was not associated with genetic susceptibility in African RA patients. Chen et al. (29) found that the *ABCB1 3,435 CC* genotype was associated with refractory RA in the Chinese population. Muralidharan et al. (11) found that Indian RA patients carrying the *T* allele of *ABCB1 3,435* had high EULAR disease activity scores, and carriers of the *CT* genotype had a higher incidence of joint deformation. The results of this study showed that there was no significant difference in the frequencies of *ABCB13435C/T* genotypes and alleles compared with the healthy controls. No statistical significance was found in the three genetic models (dominant/recessive/codominant) after adjusting for confounding factors such as age and sex, suggesting that *ABCB1 3435C/T* was not associated with susceptibility to RA. However, it is still necessary to increase the sample size and verify it in different ethnic groups.

It has been shown that the *MTHFR 677C/T* and *1298A/C* gene polymorphisms are closely related to genetic susceptibility to autoimmune diseases (30). It was found that the peripheral blood expression of *MTHFR* was significantly reduced in RA patients (31). Recent results on *MTHFR 677C/T* and *1298A/C* gene polymorphisms and RA susceptibility are highly controversial, suggesting that the effects of these polymorphisms may be closely related to region, ethnicity, or other factors. Saad et al. (12) showed that in an African American (Egyptian) population, the *MTHFR 677C/T* SNP was correlated with genetic susceptibility to RA under different genetic models (except the recessive model), and the *1298A/C* gene SNP was correlated with genetic susceptibility to RA under dominant and codominant models, while Boughrara et al. (10) reported the opposite conclusions. Inanir et al. (13) found that the *MTHFR 677C/T* SNP was associated with genetic susceptibility to RA in an Asian (Turkish) population under the dominant model and that *T* allele carriers were more susceptible to RA. Rubini et al. (32) studies in a European population showed a significantly increased susceptibility to RA in carriers of the *MTHFR 1298A/C CC* genotype; however, the *677C/T* SNP and RA susceptibility were not associated. The meta-analysis results are also controversial. Bagheri et al. (22) believe that the *MTHFR 677C/T* SNP is associated with the genetic susceptibility of African RA patients under only the recessive model, whereas in the Asian population both the *T* allele and *TT* genotype are associated with RA, and the *CC* genotype of the *1298A/C* SNP is associated with RA in the recessive model. Cen et al. (33) similar results were obtained by Bagheri et al. The research results of Yuan et al. (23) showed that *MTHFR677C/T* SNP was associated with the genetic susceptibility of RA in white people and African-Americans, while *1298A/C* SNP was not associated with the genetic susceptibility of RA. In this study, there was no significant difference in the distribution frequencies of genotypes and alleles of *MTHFR 677C/T* and *1298A/C* RA patients in Gansu, compared with healthy controls. Given the significant differences in sex composition and age distribution between the RA group and the healthy control group, adjustment for sex and age by using logistic regression analysis was performed. The *TT* genotype frequencies and *CC* genotype frequencies of *MTHFR 677C/T* were significantly different, indicating that *MTHFR 677C/T* SNP was associated with genetic susceptibility to RA. The *MTHFR 677C/T* SNP reduces the plasma *MTHFR* enzyme concentrations, which in turn affects folate metabolism and ultimately leads to the occurrence of RA. The results of this study and previous studies of the Asian RA population are basically consistent, but the results still need to be verified in larger samples of different ethnic groups and different populations.

ACPA can be produced in approximately 70 to 80% of RA patients. ACPA is an autoantibody that is specific for RA in 98% of cases. Recently, important progress has been made in elucidating the pathogenesis of ACPA-negative RA, and studies have shown that ACPA-positive and ACPA-negative RA have different genetic backgrounds and may be two different diseases (34). After applying Logistic regression analysis to adjust for gender and age, this study found that the *MTHFR 677C/T* SNP is associated with the genetic susceptibility to ACPA-positive RA. The difference *in vivo* immune disorders in patients with serum RF-negative and RF-positive RA indicates that these serological differences may indicate different subtypes of RA in the two groups, as there is a close correlation between serological subtypes and genetic factors (35). After applying Logistic regression analysis to adjust for gender and age, this study found that the *MTHFR 677C/T* SNP is associated with the genetic susceptibility to RF-positive RA.

Notably, although the *MTHFR* 677C/T polymorphism exhibited significant associations with the overall RA population and the ACPA-positive and RF-positive RA subtypes in the unadjusted multivariate analysis, these associations no longer reached statistical significance after strict Bonferroni correction for multiple testing. This suggests that the positive results observed in this study may be affected by the risk of Type I errors introduced by multiple comparisons, and the robustness of the results still requires further verification through studies with larger sample sizes.

### Study limitations and future directions

The sample size of this study is relatively limited, especially the small number of samples in the ACPA and RF subgroups, which may affect the statistical power and the reliability of subgroup analyzes. Furthermore, all samples were collected from a single center, so selection bias may exist. In the future, larger-sample, multi-center prospective studies should be conducted and validated in different ethnic groups to improve the generalizability of the results. Additional functional experiments, such as *in vitro* enzyme activity assays and animal model studies, will also help to elucidate the specific molecular mechanisms by which *MTHFR* gene polymorphisms affect folate metabolism and contribute to the pathogenesis of RA.

## Conclusion

In conclusion, this study demonstrates that the *MTHFR 677C/T* polymorphic locus may be significantly associated with susceptibility to RA and its ACPA-positive and RF-positive subtypes in the Gansu population of China, whereas the *ABCB1 3435C/T* and *MTHFR 1298A/C* polymorphisms did not show a significant association. These findings provide new ethnic-specific evidence for the genetic background of RA and offer a theoretical basis for individualized risk assessment and early intervention strategies in the future.

## Data Availability

The original contributions presented in the study are included in the article/supplementary material, further inquiries can be directed to the corresponding author.
